# Posterior airway changes during and after Herbst appliance treatment

**DOI:** 10.1007/s00784-024-06129-9

**Published:** 2025-02-05

**Authors:** Niko C. Bock, G. Sonntag, K. Klaus, S. Ruf

**Affiliations:** 1 Department of Orthodontics, University of Giessen, Schlangenzahl 14, 35392 Giessen, Germany; 2 Private Practice, Seligenstadt, Germany

**Keywords:** Posterior airway space, Class II:1, Herbst treatment, Stability

## Abstract

**Objectives:**

Herbst appliance treatment results in posterior airway space (PAS) increase. The published data, however, is based on rather small samples and shows large inter-individual variation. Therefore, the current aim was to investigate PAS changes during and after Herbst plus subsequent multibracket appliance (MBA) treatment in a retrospective cohort study and to search for possible pre-treatment influencing factors.

**Materials and methods:**

503 former Class II:1 patients (overjet = 7.8 ± 2.4 mm, ANB angle = 5.0 ± 2.1°) who had undergone treatment at 13.8 ± 3.4 years (Department for Orthodontics, University of Giessen, Germany). Cephalograms from before (T0), after 24.9 ± 9.2 months of treatment (T1) and 26.1 ± 8.0 months after treatment (T2) were analysed for PAS changes (area-size and linear distances p, t, pC2, pC3, pC4. In addition, possible influencing pre-treatment characteristics were evaluated: overjet, ANB angle, Wits appraisal, ML/NSL angle, ArGoGn angle, age and skeletal maturity.

**Results:**

On average, the PAS area increased by 23% during Herbst-MBA treatment (T1-T0) and remained constant (± 0%) thereafter (T2-T1). All linear distances also increased (6–19%) during T1-T0 and showed further increase (1–7%) during T2-T1. For all variables a large inter-individual variation existed. With regard to possible influencing factors on PAS changes, significant associations were observed for pre-treatment age and Wits appraisal of the patients.

**Conclusions:**

PAS increases during Herbst-MBA treatment. For none of the assessed variables, relapse occurred afterwards. Young age and a large Wits appraisal were determined to be beneficial for PAS enlargement.

**Clinical relevance:**

Herbst-MBA treatment seems to have a positive effect in the majority of Class II patients with reduced PAS.

**Supplementary Information:**

The online version contains supplementary material available at 10.1007/s00784-024-06129-9.

## Introduction

The posterior airway space (PAS) is part of the extrathoracic airway and is located between the posterior pharyngeal wall and the base of the tongue or soft palate [1]. A reduced PAS is often associated with obstructive sleep apnea (OSA) and snoring, the latter affecting 53% of children in the orthodontic practice [2–5].

In addition to surgical mandibular advancement, removable functional orthodontic appliances for Class II treatment such as the Activator-Headgear appliance or the Bionator have been shown to be associated with PAS enlargement of up to 12–15% both short- and long-term (linear measurements) [6, 7]. This is also true for the Herbst appliance: an investigation using two-dimensional data of a small sample size (*n* = 13) demonstrated significant enlargement of the PAS long-term (up to 37% 7 years post-treatment) when compared to untreated Class I and II controls [8]. Furthermore, short-term oropharyngeal volume changes of the Herbst appliance have been investigated in 24 Class II Herbst-MBA patients using three-dimensional cone beam computed tomography (CBCT) scans. Significant increases in PAS were found in patients treated with the Herbst appliance when compared to 20 Class I patients treated only with a MBA (up to 13% for linear and 113% for volumetric measurements) [9].

However, even if Herbst appliance treatment has been shown to result in a PAS increase [8, 10], the data in literature show a large inter-individual variation and are exclusively based on small sample sizes. Therefore, the aim of the current study was to investigate PAS changes during as well as after Herbst and subsequent MBA treatment in a large cohort of Class II:1 patients and to assess for possible pre-treatment influencing factors for these PAS changes.

## Materials and methods

### Subjects

After obtaining ethical approval (Medical Faculty, University of Giessen, Germany, No. 268/19 - amendment), the archive of the Department of Orthodontics, University of Giessen, Germany, was screened to generate the sample for this retrospective cohort study. Due to the explorative study design no sample size calculation was performed and all former Class II:1 patients, who completed active treatment with a Herbst appliance and subsequently a MBA between 1986 and 2023 including a follow-up period of at least 12 months were considered.

While no specific patient related exclusion criteria were formulated, the following criteria excluded the consideration of lateral headfilms for the present study:

- Incomplete documentation, missing lateral headfilms or lateral headfilms of insufficient quality from one of the examination time points T0, T1, or T2.
- Non-evaluable lateral headfilms due to an obviously unnatural positioning of the patient in the X-ray machine (extreme reclination or inclination of the head with the risk of influencing the depiction of the upper airways; *n* < 5 patients).

## Method

Lateral headfilms from three different time points were assessed:

- T0: before Herbst-MBA treatment.
- T1: immediately after Herbst-MBA treatment.
- T2: after a follow-up period of at least 12 months.

Due to the large time span of the study, both conventional (1986–2011) and digital (2012–2023) radiographs existed. In 287 patients, all three lateral headfilms were conventional, in 130 patients all of them were digital. In the remaining 86 patients, where part of the lateral headfilms were available in analogue and part in digital format, printouts of the digital radiographs on photo paper were obtained to ensure a standardised evaluation. For this procedure, the digital images were adjusted (reduced to 98% of the original size) to match the analogue images in terms of magnification. Subsequently, the evaluation was conducted exactly as in all other analogue lateral headfilms. This procedure as well as all radiographic assessments were performed by the same calibrated examiner (GS, co-author).

For the cephalometric measurements of analogue lateral headfilms, those were placed on a lightboard and all external light sources were eliminated; all irrelevant areas of the lightboard were covered with black cardboards and the entire room was darkened. Subsequently, all necessary cephalometric reference points and distances were marked and measured to the nearest of 0.5 mm, with double contours averaged. All digital lateral headfilms were also measured to the nearest of 0.5 mm using the ivoris^®^ analyze software (Computer konkret AG, Falkenstein/Vogtland, Germany, program version 8.2.60.100).

In addition, for all patients with digital lateral headfilms only (*n* = 130), a calculation of the PAS area was performed using the PDF-XChange Editor program (PDF-XChange Co Ltd., Chemainus, British Columbia, Canada, version 10.1.1). The mandatory calibration of the lateral headfilms for this area calculation was accomplished with the calibration tool of the PDF-XChange Editor program.

**Fig. 1:**
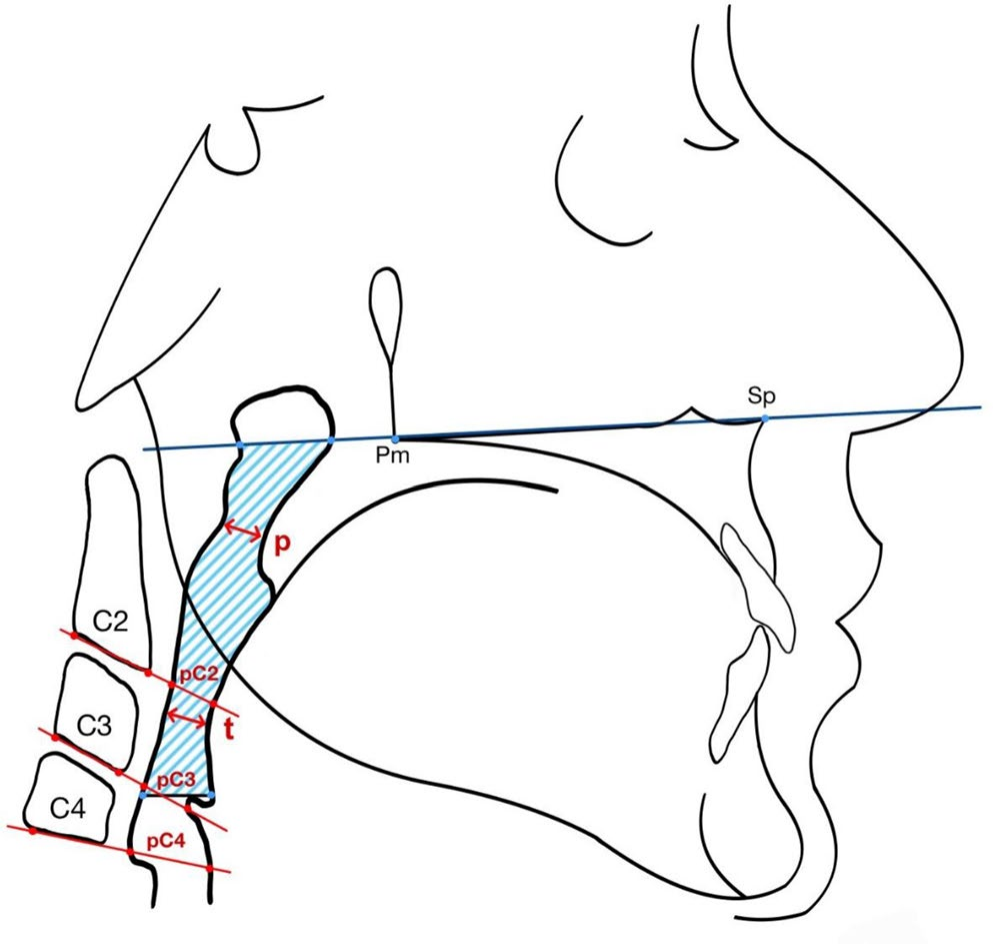
Posterior airway space (PAS): Area and linear variables (p, t, pC2, pC3 and pC4) used

The PAS area was defined as primary outcome variable. The cranial margin of the PAS area was formed by the connecting line of points Pm (intersection of the palate with the posterior contour of the maxilla) and Sp (Spina nasalis anterior), while the caudal margin was formed by the most cranially located part of the epiglottis (Fig. 1) according to boundaries previously used for the PAS [11].

In line with previous publications on the topic, the following secondary outcome parameters were measured [8, 12, 13]:

- The **distance* p*** was defined as the shortest distance between the soft palate and the posterior pharyngeal wall (Fig. 1).
- The **distance* t*** was determined as the shortest distance between the base of the tongue and the posterior pharyngeal wall (Fig. 1).
- Additionally, the **dimension of the pharynx** between the anterior and posterior walls in the plane of the caudal margin of the cervical vertebrae **C2-C4** was measured (pC2-pC4, Fig. 1).

In order to investigate potential pre-treatment influencing factors regarding changes of the PAS during Herbst and subsequent MBA therapy as well as during the follow-up period, various cephalometric distances and angles commonly used in orthodontics were also measured. These parameters include overjet (OJ), Wits appraisal, ANB angle, ML/NSL angle and ArGo’Gn angle. In addition, age and skeletal maturity (C3-stage according to Hassel and Farman [14]) were determined. The respective skeletal maturity stages were pooled into the groups “pre-peak”, “peak” and “post-peak”.

### Statistical method

To assess the reliability of the measurements, the lateral cephalometric radiographs of 20 patients with all radiographs in digital form and 20 patients with all radiographs in analogue form were measured a second time by the same examiner with a time interval of at least 12 weeks between the measurements. Subsequently, the intraclass correlation coefficient (ICC, two-way random, consistent, single measures) was determined. All measured primary and secondary outcome parameters showed very strong consistency at all three examination time points (*r* > 0.91). In addition, for 10 patients with completely analogue radiographs those were digitized and re-evaluated digitally to assess kind of an inter-method error. The respective intraclass correlation coefficient was *r* > 0.88. The same value was determined when another examiner, who is also familiar with the method, reassessed the radiographs of 10 patients.

Furthermore, the method error was calculated using Dahlberg’s formula and resulted in values between 1.2 mm² and 1.3 mm² for area measurements, 0.1 mm and 0.2 mm for linear measurements and 0.1° and 0.3° for angular measurements.

The data accumulation was performed using MS Excel 2007 (Microsoft, Redmond, Washington, USA) and the statistical analysis was performed using the software SPSS Statistics (IBM, Armonk, USA, Version 29); p-values ≤ 0.05 were considered statistically significant.

All data showed sufficient approximation to a normal distribution, allowing the calculation of arithmetic means. Descriptive statistics were computed for all variables of interest at all time points. A paired t-test was used to test for significant changes between examination time points. Spearman correlation was used to examine relationships between various primary and secondary outcome parameters.

The parameter PAS area was only determined for the 130 patients for whom digital lateral headfilms were available at all three time points. In addition, the distance pC4 could only be measured for 207 patients of the cohort due to missing or incomplete depiction of the vertebrae C4 on the lateral headfilm. To ensure that these two subgroups of 130 and 207 patients, respectively, did not differ from the remaining patients included in the study, t-tests for equality of means were calculated for all time periods (Supplementary Table 1).

Similarly, t-tests were conducted between those patients with monognathic retention appliances only (*n* = 376) and those with a bignathic retention appliance or a combination of mono- and bignathic devices (*n* = 127) to determine whether a difference existed regarding the PAS changes during the follow-up period (T2-T1) (Supplementary Table 1).

All statistical analyses were conducted by an external independent statistician.

## Results

In total, 503 patients (268 female and 235 male) were included in the study. The average age of the patients at the start of treatment was 13.8 ± 3.4 years. The period of active treatment (T1-T0) took on average 24.9 ± 9.2 months, Herbst phase: 8.4 ± 5.6 months; MBA phase: 16.5 ± 7.2 months), and the post-treatment observation period (T2-T1) was 26.1 ± 8.0 months.

The mean values, standard deviations, medians, minima and maxima of the measured variables at all three points of observation are presented in Table 1 (Supplementary Table 2 contains these data divided by pre-treatment skeletal maturity). Additionally, Table 2 shows the changes of all assessed variables for the observation periods T1-T0, T2-T1 and T2-T0.

### PAS changes: area (*n* = 130)

**Fig. 2:**
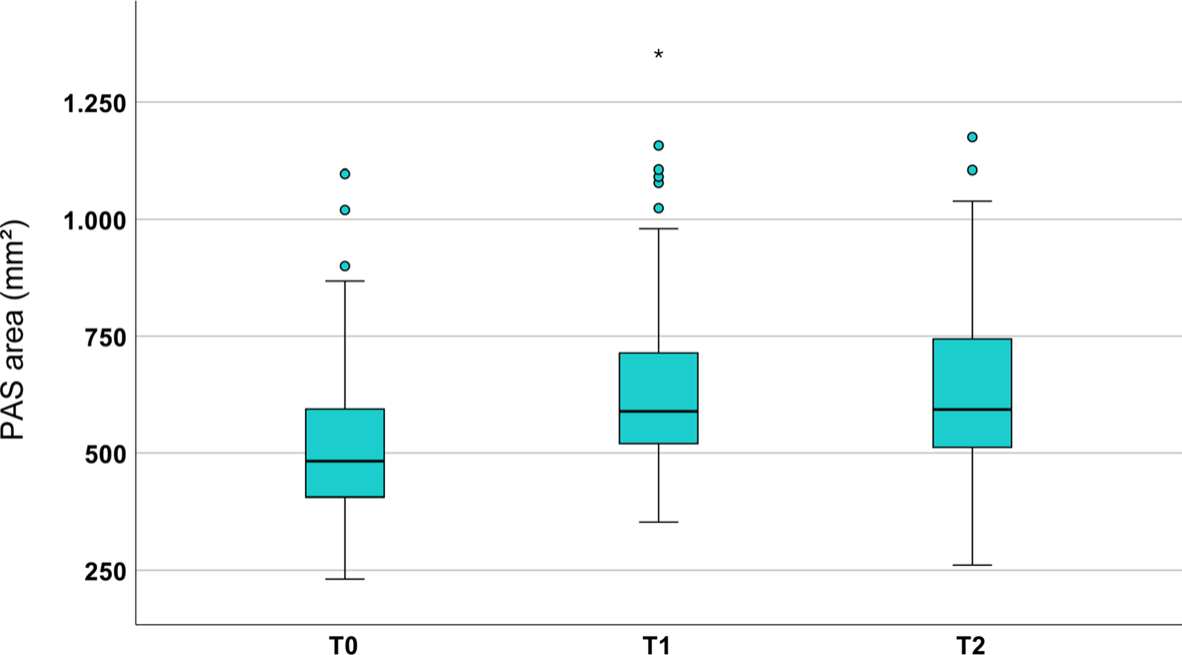
Boxplots exhibiting the values obtained for PAS area at T0, T1 and T2 (*n*=130)

**Fig. 3:**
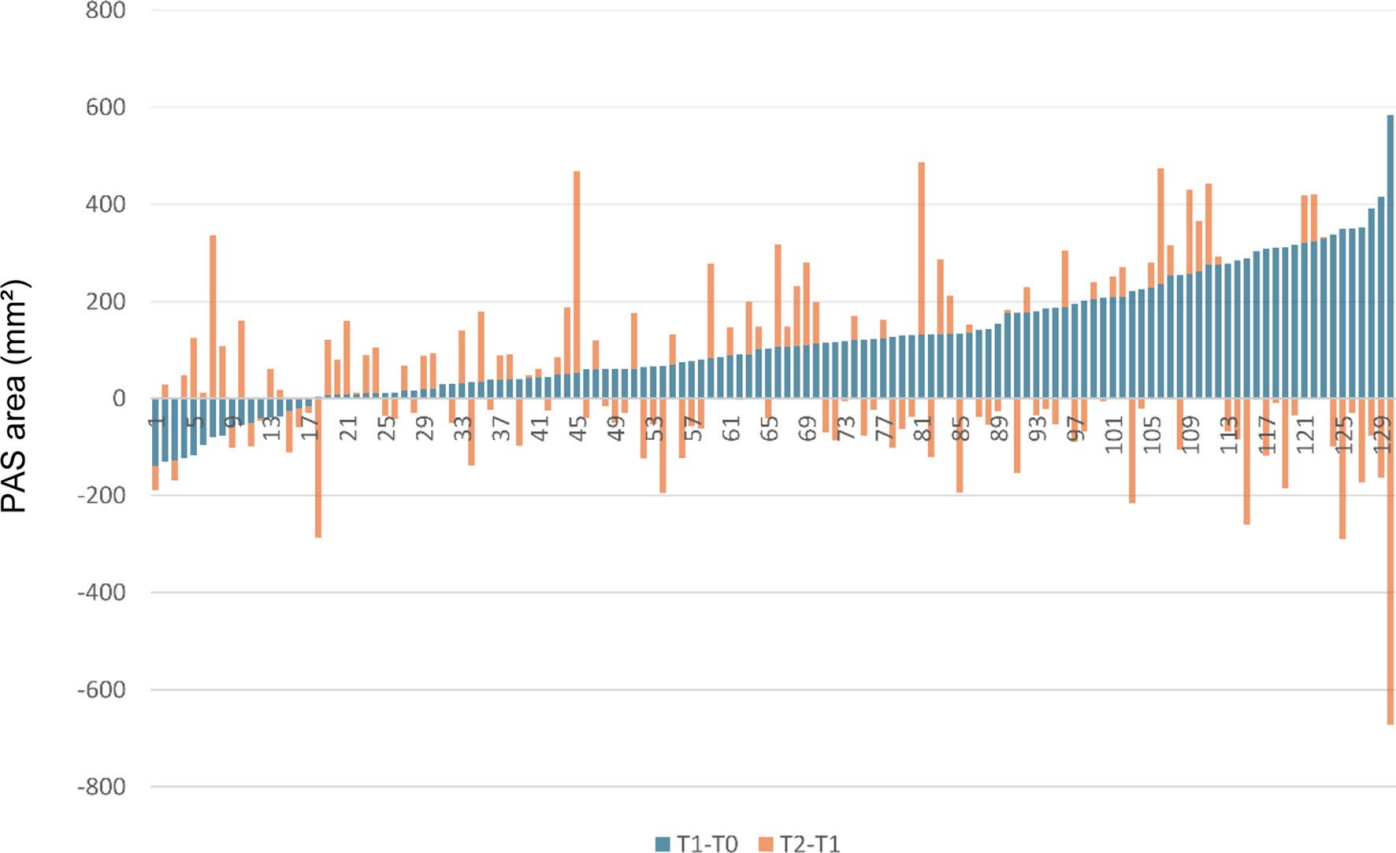
PAS area changes during Herbst-MBA treatment (T1-T0) and the post-treatment period (T2-T1) (*n*=130)

Before treatment (T0), the PAS area had a mean size of 514.1 ± 153.22 mm². During Herbst-MBA therapy it increased by 22.7 ± 25.3% (117.0 ± 129.65 mm²; *p* < 0.001) and remained relatively constant during the post-treatment period (0.0 ± 25.0%; 0.5 ± 128.44 mm²; *p* = 0.962). Thus, an overall average enlargement of the PAS area of 22.9 ± 28.8% (117.5 ± 147.90 mm²; *p* < 0.001) was present after Herbst-MBA therapy and an average post-treatment observation period of 26 months (Figs. 2 and 3; Tables 1 and 2).

**Table 1:**
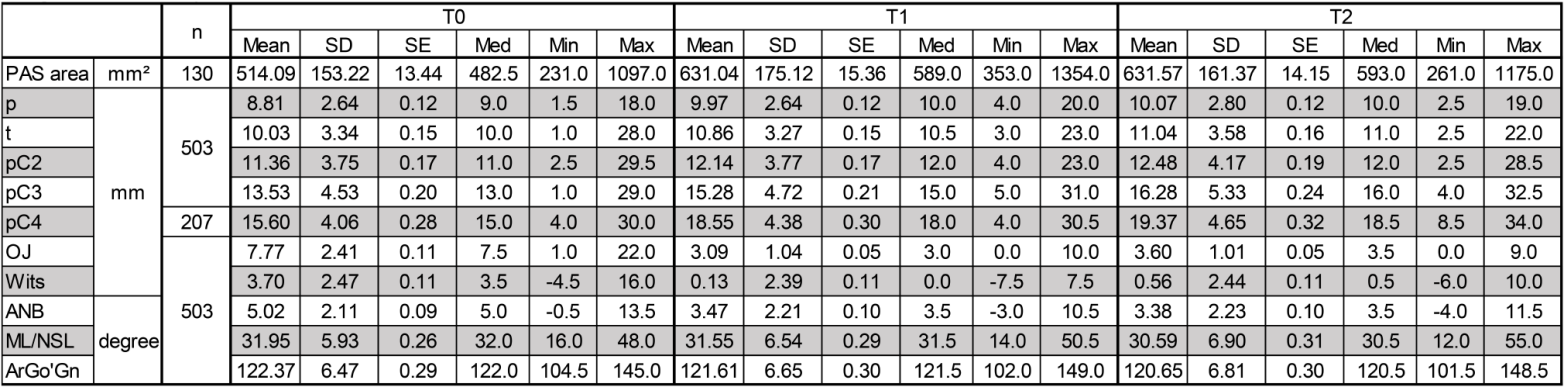
Cephalometric measurements: number of subjects (n), means, standard devations (SD), standard errors (SE), medians (Med), minima (Min) and maxima (Max) of all variables are given for the time points T0, T1, and T2

**Table 2:**
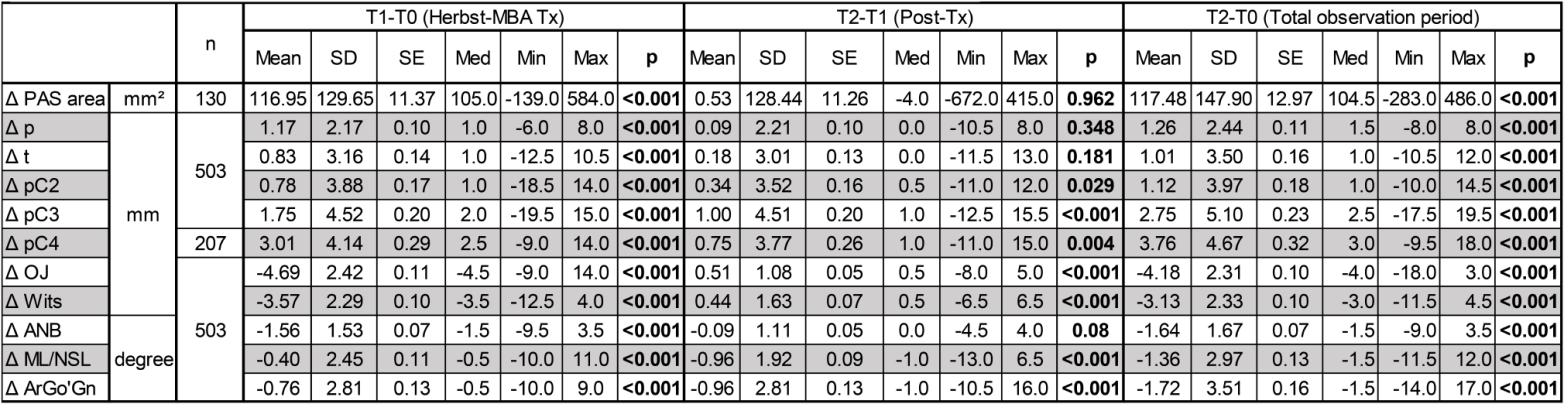
Cephalometric measurements: number of subjects (n), means, standard devations (SD), standard errors (SE), medians (Med), minima (Min) and maxima (Max) of all variables are given for the time points T1-T0, T2-T1 and T2-T0 as well as the *p*-values of the respective changes

### PAS changes: linear distances (*n* = 503)

During Herbst-MBA therapy (T1-T0), distance p increased by 12.5 ± 25.0% (1.2 ± 2.17 mm; *p* < 0.001). Post-treatment (T2-T1), there was some additional increase by 1.1 ± 25.0% (0.1 ± 2.21 mm; *p* ≥ 0.05).

Similarly, distance t showed an increase by 8.0 ± 32.0% (0.8 ± 3.16 mm; *p* < 0.001) during Herbst-MBA therapy and remained stable during the post-treatment period (+ 2.0 ± 30.0%; 0.2 ± 3.01 mm; *p* ≥ 0.05).

**Fig. 4:**
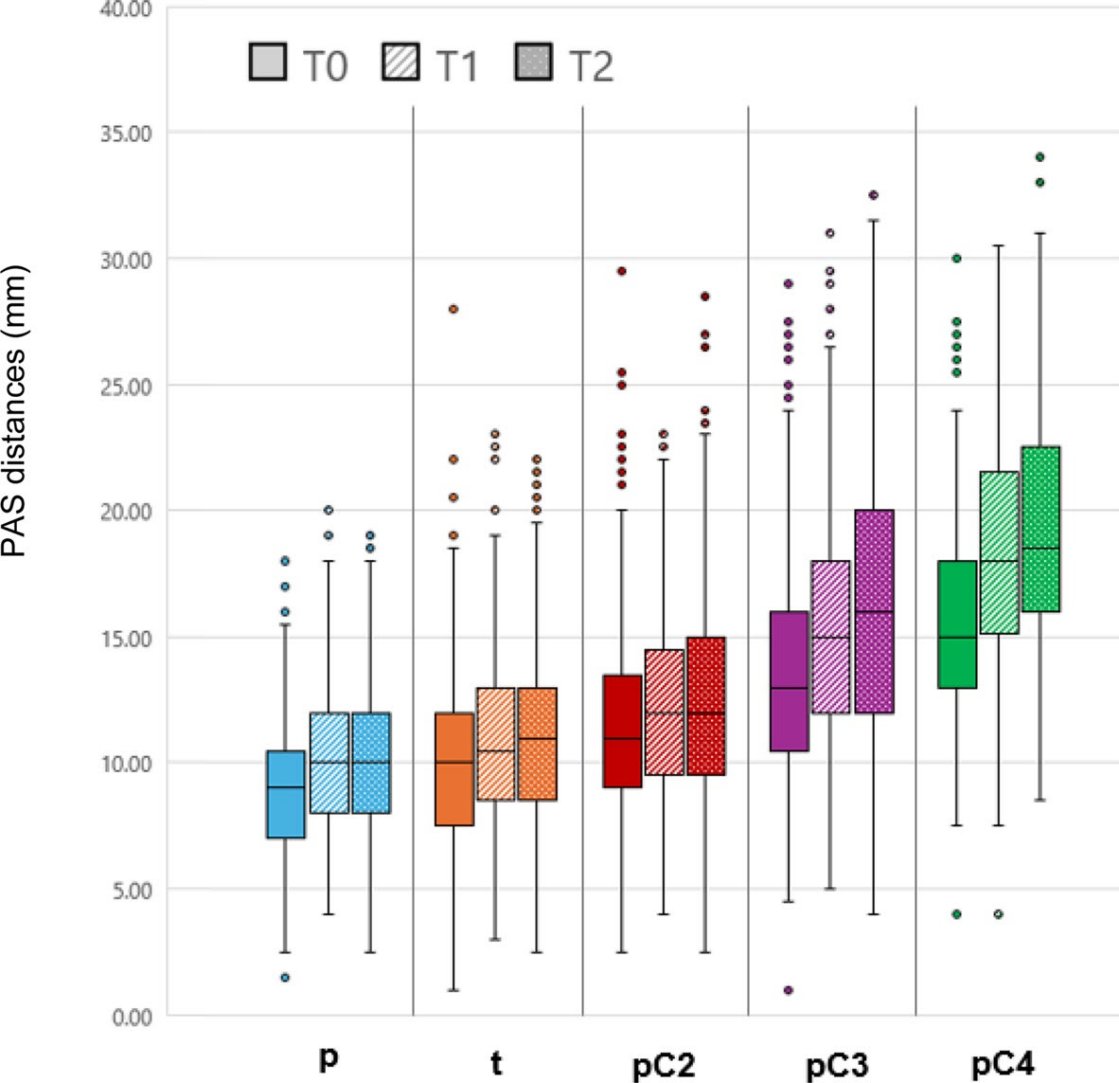
Boxplots exhibiting the data obtained for the PAS linear changes p, t, pC2, pC3 and pC4 at T0, T1 and T2 (*n* = 503)

Enlargement was also observed in the region of the cervical vertebrae C2-C4. Accordingly, distance pC2 increased by 7.0 ± 34.2% (0.8 ± 3.88 mm; *p* < 0.001), distance pC3 by 13.3 ± 33.3% (1.8 ± 4.52 mm; *p* < 0.001) and distance pC4 by 19.2 ± 26.3% (3.0 ± 4.14 mm; *p* < 0.001) during Herbst-MBA therapy (T1-T0). For all three parameters, some further increase (*p* ≥ 0.05) was measured post-treatment (T2-T1) (pC2: 2.6 ± 30.7%; 0.3 ± 3.52 mm; pC3: 7.4 ± 33.3%;1.0 ± 4.51 mm; pC4: 5.1 ± 30.1%; 0.8 ± 3.77 mm; Fig. 4).

However, for all investigated variables a large interindividual variability was observed, which can be seen on the basis of the minima and maxima given in Tables 1, 2 and 3 as well as in Figs. 3 and 4.

**Table 3:**
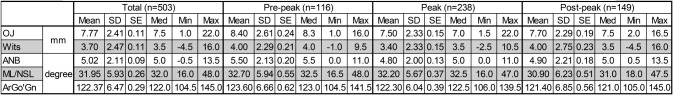
Pre-treatment (T0) cephalometric measurements divided by skeletal maturity groups “total”, “pre-peak”, “peak” and “post-peak”: means, standard deviations (SD), standard errors (SE), medians (Med), minima (Min) and maxima (Max) of overjet, wits appraisal, ANB angle, ML/NSL angle and ArGo’Gn angle are given

### Possible pre-treatment influencing factors

With regard to possible pre-treatment influencing factors, a small but significant association (*r* = 0.227; *p* = 0.009) was determined for the increase of the PAS area during active therapy (T1-T0) and the pre-treatment Wits appraisal (T0), indicating that patients with a larger Wits appraisal before therapy showed greater changes in PAS area during therapy.

Furthermore, a significant negative association (*r*=-0.294; *p* < 0.001) was observed between the changes of the linear distance pC4 during active therapy (T1-T0) and the pre-treatment age of the patients (T0), implying that smaller changes were observed in older patients.

When comparing the data of the 130 patients where digital lateral headfilms were available and thus, PAS area measurements could be performed, to the remaining patients included in the study, statistically significant differences (*p* < 0.05) were seen for linear distances t and pC2 (T1-T0) and linear distance pC3 (T2-T1), showing larger changes in the 130 patients with digital lateral headfilms (Supplementary Table 1). This difference could however in part be explained by the small difference in magnification (2%). For the 207 patients where the distance pC4 could be measured, no statistically significant differences (*p* = 0.111–0.991) existed when comparing their data (T1-T0 and T2-T1) to the remaining patients (Supplementary Table 1).

The post-treatment changes (T2-T1) in patients with monognathic respectively bignathic retention appliances did not differ (*p* = 0.384–0.756; Supplementary Table 1).

## Discussion

503 patients included in the present retrospective cohort study exhibited the same malocclusion pre-treatment and they all underwent a similar therapy. To the best of our knowledge, this is the largest sample size regarding the examination of PAS changes during and after Class II:1 Herbst-MBA therapy so far. In addition, data on PAS area measurements in orthodontics are scarce. It is however important to consider that not all patients exhibited the same severity of Class II:1 malocclusion (mean pre-treatment overjet: 7.8 ± 2.4 mm; ANB: 5.0 ± 2.1°) (Table 3).

The radiographs had been generated over a period of more than 35 years, therefore both conventional and digital lateral headfilms were included. In addition, no information on Body Mass Index was available for the majority of patients and could thus not be used as a discriminating factor. While the patient cohort also differed in terms of skeletal maturity, the three skeletal maturity groups - when compared - exhibited a similar within-group-variation regarding pre-treatment malocclusion severity (Table 3).

As the majority of patients exhibited a certain amount of physiological remaining growth during therapy (pre-peak: *n* = 116; peak = 238; post-peak: *n* = 149), it cannot be determined which changes were actually attributable to the therapeutic measures and which to the physiological growth of the patients. All PAS measurement data separated according to skeletal maturity, however, are given in Supplementary Table 2. Unfortunately, no untreated control group was available for ethical reasons in terms of radiation protection.

Another limitation of the present study is the evaluation of two-dimensional lateral headfilms for assessing a three-dimensional anatomical space. While the lateral headfilm provides information about the pharyngeal airway in the sagittal and vertical dimensions, no statement can be made regarding the transverse plane. Consequently, this also applies to the actual volume of the PAS, limiting the overall interpretability [15]. It has often been discussed that the accurate assessment of the pharynx is limited by potential physiological deformations caused by adjacent structures or the physiologically oval cross-section of the primarily muscular pharynx [16]. Therefore, the realistic assessment of the PAS through two-dimensional radiographs is compromised as anatomically related obstructions of the pharyngeal airway can only be clearly depicted through three-dimensional imaging [17].

The validity of lateral headfilm measurements in the assessment of PAS was analysed in the context of apnea therapy with protrusion splints [18]. The actual lumen, measured using magnetic resonance imaging (MRI), was on average 11–12% smaller than the linear values in the lateral cephalometric radiograph suggested. They observed a linear correlation between the results in the lateral headfilms and those in the MRIs, indicating that even if the assessment of absolute values is limited, the relative assessment of longitudinal changes is possible.

Furthermore, the results of another investigation also demonstrate that there are no basic differences between the dimension of the PAS changes in two-dimensional compared to three-dimensional imaging [19]. The research group examined both CBCTs and lateral headfilms after bignathic osteotomy in Class II patients. Immediately after the intervention, they measured an average PAS enlargement of 41.6% in the CBCTs and 46.8% in the lateral headfilms.

It is also worth to mention, that even if three-dimensional imaging offers diagnostic advantages, it may not always be feasible in clinical practice due to guidelines in terms of radiation protection [20]. For instance, obtaining lateral headfilms at various time points is a standard procedure in orthodontic diagnostics and treatment [21], which allows the longitudinal examination of such a large patient cohort for this study. Such an investigation would not have been possible using data from three-dimensional imaging for the reasons mentioned above. Furthermore, the evaluation of lateral headfilms has been the standard method in all studies regarding the orthodontic influence on the PAS so far.

Factors affecting the accuracy of PAS measurements in both two- and three-dimensional radiographs are posture, breathing and other dynamic conditions of the patient. This is of particular relevance as it takes several seconds to acquire the respective image (up to 17 s for three-dimensional radiographs) and it cannot be ensured that patients neither breathe nor swallow during the time of exposure which potentially influences the dimension of the PAS [22].

The variables used for PAS assessment in the current study represent a selection from several investigations in the literature [8, 11–13]. However, a large variety of other possible PAS measurements on different pharyngeal levels exists, but so far there is a lack of consensus regarding their reliability and validity.

Due to the large sample size several changes exhibit statistical significance, even if the standard deviation appears to be rather large in comparison to the actual mean value; to make these data more plausible, the values of the standard error are given in the respective tables.

In the present sample with an average age of 13.8 years at the start of treatment, a significant enlargement of the PAS area (22.7 ± 25.3%) was seen during the 2.1 years of Herbst-MBA therapy, which remained stable after treatment. While there are no comparable data from Herbst-MBA treatment, a study investigating the influence of a Headgear-Activator on the PAS area in a group of 64 patients with an average age of 10.4 years determined a significant increase of 36.6%, however over a longer period of 4.4 years of orthodontic therapy [6]. This difference (+ 22.7% vs. +36.6%) might be explained by the younger pre-treatment age and longer treatment duration/natural growth in the Headgear-Activator. In addition, a slight difference exists regarding the method of the two investigations in terms of the boundaries defining the measured PAS area. What is more difficult to explain, however, is the difference regarding the post-treatment development. In the Headgear-Activator group, further increase was seen, amounting to a total of 62.6% (278.7 mm²) for the total observation period of 7.6 years (difference of radiographic magnification 0.5%). The corresponding value of the present study is distinctly smaller (22.9%, 117.5 mm²), but was measured after a shorter period of 4.3 years. Nevertheless, the final values of both investigations (724.2 mm² after Headgear-Activator therapy and 631.6 mm² after Herbst-MBA therapy) showed a large difference, which cannot fully be explained by the difference in pre-treatment age with the corresponding growth potential and the duration of active therapy. Nevertheless, the age of the patients at the end of the observation period (around 18 years in both groups) suggested that no substantial further improvement can be expected. Maybe the additional use of the Headgear increased the effects on the PAS when compared to Herbst-MBA treatment as it has been discussed that maxillary growth inhibition might lead to an increased anterior rotation of the mandible, potentially resulting in additional PAS enlargement.

In another study, the linear distances p and t exhibited significant increases of 0.6 mm vs. 0.2 mm, respectively, during Herbst treatment [8]. The latter authors observed further increase of the linear distances during a follow-up period of 6 years (on average) resulting in a total increase of 2.3 mm of distance p and 3.3 mm of distance t. In comparison to the present study (p: +1.3 mm; t: +1.0 mm), larger overall changes were observed by these investigators. However, also in this study the patients were younger at the start of treatment (mean age: 12.4 years vs. 13.8 years).

All distances pC2, pC3 and pC4 showed increases during Herbst-MBA therapy (pC2: +0.8 mm; pC3: +1.8 mm, pC4: +3.0 mm) in the present study and remained stable after therapy. The same parameters were also used for assessing PAS changes in a sample of Herbst patients aged 13.7 years [13], where the authors observed increases in distances during therapy by 0.9 mm for pC2, 1.4 mm for pC3 and 0.8 mm for pC4.

The enlargement of the pharyngeal airway during Herbst and subsequent MBA therapy is also evident in CBCT scans [9]. Overall, a volume enlargement of 113.6% was observed during Herbst-MBA treatment. In this study, patients were on average 11.6 years old at the start of therapy and thus, significantly younger than in the present study. In the present study, a PAS area enlargement of 22.9% compared to the baseline (T0) was observed during Herbst-MBA therapy and the follow-up period. A possible explanation for this difference could be that the advancement of the mandible leads to an enlargement of the PAS, particularly in the lateral area [23, 24]. This change is difficult to be seen in lateral headfilms, although it can be measured in three-dimensional imaging.

As Herbst appliance therapy naturally results in mandibular advancement [25, 26], this must be considered as the main reason for PAS enlargement during treatment. However, additional PAS enlargement might occur due to treatment-induced morphological changes of the lower jaw. After Herbst appliance treatment, patients tend to exhibit a decrease of the ML/NSL angle and an increase of the lower posterior face height when compared to untreated control individuals [27]. This suggests a modification of the post-treatment growth pattern towards a more pronounced counterclockwise rotation of the mandible, which might affect the PAS positively [8].

Furthermore, appraising the current results, it is important to consider the large interindividual variation observed for all variables. Similar observations were also made in several other studies [8, 12, 22], indicating that predicting the individual impact of Class II treatment on the PAS seems impossible. Nevertheless, it has been shown in the current investigation that on average all PAS variables increase during Class II:1 treatment with a Herbst-MBA appliance. Whether this average increase differs from Class I patients or to what extent it has a positive functional effect on patient breathing short or long-term is currently unknown and should be investigated in future.

## Conclusions

PAS increases in terms of area and linear distance during Herbst plus subsequent MBA treatment. Even if relapse wasn’t seen for any of the assessed variables according to the average values, a large interindividual variation existed. In terms of possible pre-treatment influencing factors, it was determined that a young patient age and a large Wits appraisal have a positive effect on PAS enlargement during Herbst-MBA treatment.

## Electronic supplementary material

Below is the link to the electronic supplementary material.

Supplementary Material 1

Supplementary Material 2

## Data Availability

No datasets were generated or analysed during the current study.
