# Supplementary material for: Posterior airway changes during and after Herbst appliance treatment
Source: Clin Oral Investig. 2025 Feb 5;29(2):114. doi: 10.1007/s00784-024-06129-9 (PMC11865216; doi:10.1007/s00784-024-06129-9)
Supplement: Supplementary file 1 — Supplementary Material 1 [file 784_2024_6129_MOESM1_ESM.pdf]

Supplementary Table 1

P-values for the comparison of those patients where the PAS area respectively the distance pC4 was measured to the remaining patients included in the study (n=130/n=207 vs. n=373/n=296) as well as for the comparison of those with monognathic and bignathic retention appliances (n=376 vs. n=127). For the parameter PAS area, where occasional significant differences were observed, the means and standard deviations (SD) of the two groups for the periods T1-T0 and T2-T1 are also provided.

		**PAS area**										**Distance pC4**		**Retention appliance**
		**measureable (n=130) vs. not measurable (n=373)**										**measurable (n=207) vs. not measurable (n=296)**		**monognathic (n=376) vs. bignathic (n=127)**
		**T1-T0**					**T2-T1**					**T1-T0**	**T2-T1**	**T2-T1**
			**n=130**		**n=373**			**n=130**		**n=373**				
		p	Mean	SD	Mean	SD	p	Mean	SD	Mean	SD	p	p	p
PAS area	mm²											0.991	0.160	0.735
p	mm	0.132	1.42	1.97	1.08	2.24	0.268	-0.09	2.27	0.16	2.18	0.219	0.111	0.384
t	mm	0.003	1.53	2.72	0.58	3.27	0.095	-0.20	2.87	0.31	3.05	0.523	0.790	0.756
pC2	mm	0.005	1.59	3.13	0.50	4.08	0.057	-0.16	3.03	0.52	3.66	0.160	0.535	0.611
pC3	mm	0.108	2.30	3.69	1.56	4.77	0.034	0.28	3.87	1.25	4.70	0.329	0.803	0.598
pC4	mm	0.138	4.16 (n=25)	2.86	2.85 (n=182)	4.27	0.785	0.56 (n=25)	2.69	0.78 (n=182)	3.90			0.751
