# Supplementary material for: Posterior airway changes during and after Herbst appliance treatment
Source: Clin Oral Investig. 2025 Feb 5;29(2):114. doi: 10.1007/s00784-024-06129-9 (PMC11865216; doi:10.1007/s00784-024-06129-9)
Supplement: Supplementary file 2 — Supplementary Material 2 [file 784_2024_6129_MOESM2_ESM.pdf]

Supplementary Table 2

Cephalometric measurements divided by skeletal maturity groups “pre-peak”, “peak” and “post-peak”: number of subjects (n), means, standard deviations (SD), standard errors (SE), medians (Med), minima (Min) and maxima (Max) of PAS area, p, t, pC2, pC3 and pC4 are given for the time points T0, T1, and T2.

T0		n	pre-peak (n=116)						n	peak (n=238)						n	post-peak (n=149)					
			Mean	SD	SE	Med	Min	Max		Mean	SD	SE	Med	Min	Max		Mean	SD	SE	Med	Min	Max
PAS area	mm²	45	422.18	99.40	14.82	423.0	231.0	731.0	56	541.04	160.89	21.50	520.0	310.0	1097.0	29	604.69	135.12	25.09	581.0	387.0	900.0
p	mm	116	7.75	2.48	0.23	8.0	1.5	16.0	238	8.75	2.71	0.18	8.5	3.0	17.0	149	9.72	2.33	0.19	9.5	2.5	18.0
t			8.93	3.32	0.31	8.5	2.5	22.0		10.02	3.23	0.21	10.0	1.0	20.5		10.92	3.27	0.27	11.0	4.5	28.0
pC2			10.00	3.60	0.33	10.0	2.5	25.0		11.50	3.82	0.25	11.5	3.0	25.5		12.19	3.49	0.29	12.0	5.0	29.5
pC3			11.55	4.13	0.38	11.5	4.5	25.0		13.30	4.32	0.28	13.0	1.0	27.5		15.43	4.43	0.36	15.0	5.0	29.0
pC4		41	12.67	2.89	0.45	13.0	7.5	20.0	93	14.95	3.36	0.32	15.0	4.0	27.5	73	18.23	4.11	0.48	17.0	10.5	30.0

T1		n	pre-peak (n=116)						n	peak (n=238)						n	post-peak (n=149)					
			Mean	SD	SE	Med	Min	Max		Mean	SD	SE	Med	Min	Max		Mean	SD	SE	Med	Min	Max
PAS area	mm²	45	569.56	133.25	19.86	564.0	370.0	896.0	56	663.11	194.70	26.02	607.0	353.0	1354.0	29	664.52	172.15	31.97	620.0	475.0	1157.0
p	mm	116	9.03	2.49	0.23	9.0	4.0	18.0	238	10.21	2.69	0.17	10.0	4.0	20.0	149	10.33	2.50	0.21	10.5	4.5	18.0
t			9.57	2.84	0.26	9.5	4.0	17.0		11.07	3.25	0.21	10.5	3.0	23.0		11.54	3.34	0.27	11.5	4.5	22.5
pC2			10.66	3.30	0.31	10.5	5.5	20.5		12.35	3.74	0.24	12.0	4.0	23.0		12.96	3.85	0.32	13.0	5.5	23.0
pC3			13.44	4.15	0.39	13.5	6.0	26.0		15.51	4.74	0.31	15.5	5.0	29.5		16.36	4.72	0.39	16.0	6.0	31.0
pC4		41	17.43	3.49	0.55	17.0	12.5	24.5	93	18.18	3.92	0.38	18.0	8.0	29.0	73	19.85	5.25	0.66	19.5	4.0	30.5

T2		n	pre-peak (n=116)						n	peak (n=238)						n	post-peak (n=149)					
			Mean	SD	SE	Med	Min	Max		Mean	SD	SE	Med	Min	Max		Mean	SD	SE	Med	Min	Max
PAS area	mm²	45	581.98	136.59	20.36	570.0	261.0	1039.0	56	664.05	174.71	23.35	652.0	356.0	1175.0	29	645.79	156.29	29.02	588.0	433.0	1007.0
p	mm	116	9.40	2.70	0.25	9.0	3.0	18.0	238	10.29	2.92	0.19	10.0	2.5	18.5	149	10.23	2.61	0.21	10.0	5.0	19.0
t			10.22	3.45	0.32	10.0	2.5	20.5		11.37	3.65	0.24	11.0	4.0	22.0		11.15	3.47	0.28	11.0	4.5	22.0
pC2			11.49	4.10	0.38	11.0	2.5	24.0		12.84	4.16	0.27	12.5	4.0	28.5		12.69	4.15	0.34	12.5	4.5	27.0
pC3			14.74	5.07	0.47	14.0	6.0	32.5		16.58	5.30	0.34	16.5	4.0	30.5		17.01	5.38	0.44	16.0	4.5	31.5
pC4		41	19.54	4.52	0.71	19.0	13.5	30.0	93	18.44	4.21	0.41	18.0	10.0	33.0	73	20.81	5.09	0.64	20.0	8.5	34.0
